# Are health systems interventions gender blind? examining health system reconstruction in conflict affected states

**DOI:** 10.1186/s12992-018-0401-6

**Published:** 2018-08-30

**Authors:** Valerie Percival, Esther Dusabe-Richards, Haja Wurie, Justine Namakula, Sarah Ssali, Sally Theobald

**Affiliations:** 1 International Affairs, Norman Paterson School of International Affairs, Carleton University, 5319 Richcraft Building, 1125 Colonel By Drive, Ottawa, ON K1S 5B6 Canada; 2 University of Leeds, Leeds, UK; 3 ReBUILD Research Consortium, College of Medicine and Allied Health Sciences, University of Sierra Leone, Freetown, Sierra Leone; 4 ReBUILD Consortium, School of Public Health, Makerere University, Kampala, Uganda; 5 School of Women and Gender Studies, ReBUILD consortium, Makerere University, Kampala, Uganda; 6 Social Science and International Health, ReBUILD and RinGs Consortium, Liverpool School of Tropical Medicine, Liverpool, UK

**Keywords:** Gender and development, Gender and health, Health systems, Post-conflict

## Abstract

**Background:**

Global health policy prioritizes improving the health of women and girls, as evident in the Sustainable Development Goals (SDGs), multiple women’s health initiatives, and the billions of dollars spent by international donors and national governments to improve health service delivery in low-income countries. Countries recovering from fragility and conflict often engage in wide-ranging institutional reforms, including within the health system, to address inequities. Research and policy do not sufficiently explore how health system interventions contribute to the broader goal of gender equity.

**Methods:**

This paper utilizes a framework synthesis approach to examine if and how rebuilding health systems affected gender equity in the post-conflict contexts of Mozambique, Timor Leste, Sierra Leone, and Northern Uganda. To undertake this analysis, we utilized the WHO health systems building blocks to establish benchmarks of gender equity. We then identified and evaluated a broad range of available evidence on these building blocks within these four contexts. We reviewed the evidence to assess if and how health interventions during the post-conflict reconstruction period met these gender equity benchmarks.

**Findings:**

Our analysis shows that the four countries did not meet gender equitable benchmarks in their health systems. Across all four contexts, health interventions did not adequately reflect on how gender norms are replicated by the health system, and conversely, how the health system can transform these gender norms and promote gender equity. Gender inequity undermined the ability of health systems to effectively improve health outcomes for women and girls. From our findings, we suggest the key attributes of gender equitable health systems to guide further research and policy.

**Conclusion:**

The use of gender equitable benchmarks provides important insights into how health system interventions in the post-conflict period neglected the role of the health system in addressing or perpetuating gender inequities. Given the frequent contact made by individuals with health services, and the important role of the health system within societies, this gender blind nature of health system engagement missed an important opportunity to contribute to more equitable and peaceful societies.

## Background

Improving the health of women and girls is a global development priority. Sustainable Development Goal (SDG) Three “to ensure healthy lives and promote well-being,” includes specific targets to reduce maternal mortality and ensure universal access to reproductive health services. Yet health indicators of women and girls, particularly maternal mortality and HIV rates among adolescent girls and adult women, remain problematic in many low-income countries, while the global burden of non-communicable diseases (NCDs) among women is rising. These indicators reflect socio-economic contexts of pervasive gender inequalities as well as the failure of domestic health systems to effectively address and improve women’s health [1]. International development initiatives direct resources to women and girls’ health to decrease maternal mortality, strengthen reproductive rights, and address the female burden of HIV/AIDS. The donor community commits billions of dollars to support these initiatives; the Institute for Health Metrics and Evaluation (IHME) estimated that development assistance focused on maternal health exceeded 3.8 billion USD in 2016 [2].

The SDGs also include a clear commitment to achieve gender equality and empower all women and girls. Yet despite some lip service [3], the global health community has not incorporated gender equity as a goal of its health interventions, nor has it explicitly made the connection between health system attributes and gender equity [4]. For example, the 2015 Lancet Commission on Women and Health called for women’s access to Universal Health Care (UHC), and outlined how gender inequities are undermining the effectiveness and efficiency of health care services [1], but neglected to examine how health systems themselves can perpetuate and exacerbate gender inequities.

Global development and health researchers have not explored the extent to which health system engagement contributes to gender equity, nor if and how gender considerations are integrated into efforts by both international and domestic stakeholders to strengthen health systems, ensure they are gender equitable, and contribute to broader gender equality within society. There is no explicit definition of a gender equitable health system, nor a description of the attributes of gender equity within the various components of health systems [4]. Most research on both health systems as well as specific health sector interventions, such as maternal health, assumes that health systems are gender neutral – if you strengthen the health system, it will be able to effectively and equitably address the health needs of men, women, girls and boys. Yet our research suggests otherwise: health systems are embedded in and shaped by their social context, and the health system will reflect and reinforce social norms surrounding women, girls, men and boys [4].

This paper begins the process of exploring the relationship between health system engagement and gender equity. It examines this relationship by analyzing health system interventions in countries emerging from violence and conflict. The post-conflict period provides a unique opportunity to analyse health reforms and gender equity. Donor resources are often readily available to ‘build it back better,’ which includes gender responsive health systems. Political barriers may be temporarily absent, and social norms regarding gender may be more fluid and adaptable in the immediate time period following violence. To explore if and how health systems incorporate gender, we examined four diverse contexts of post-conflict health engagement - Mozambique, Timor Leste, Sierra Leone and Northern Uganda – to analyse if and how the rebuilding of health systems addresses gender equity.

## Methods

**Fig. 1:**
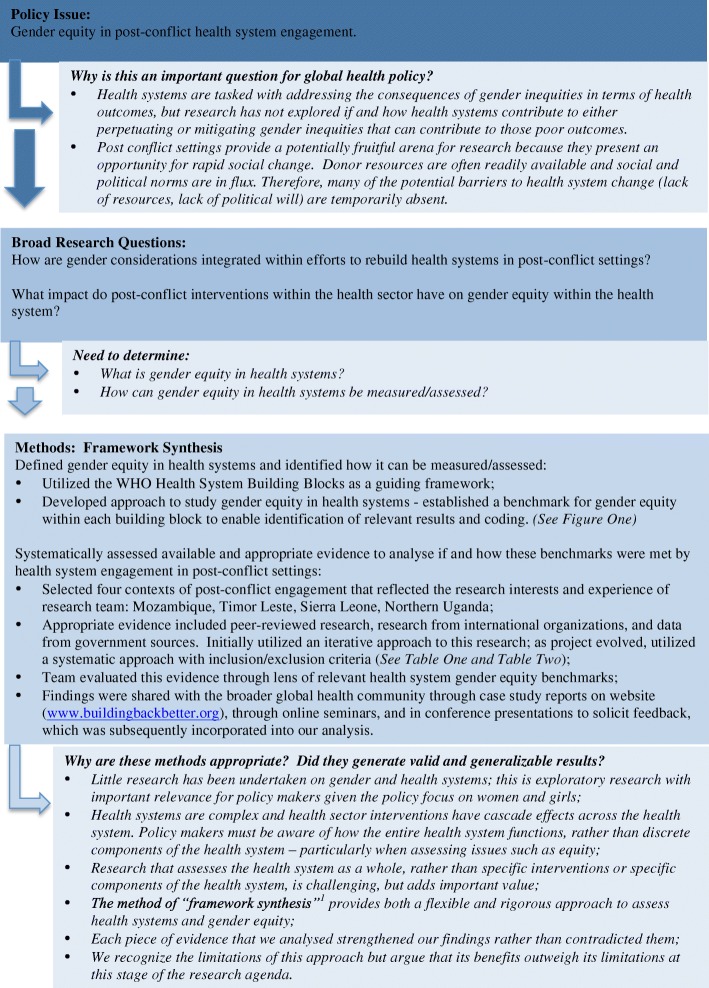
Research Approach

Billions of dollars are being spent on interventions that utilize health systems to deliver services to improve the health of women and girls without questioning if health systems themselves are part of the problem. It is critically important to inject rigorous evidence on the effectiveness of these health systems while they are being rebuilt. Our research approach is summarized in Fig. 1. The research team was guided by the following questions: How are gender considerations integrated within the efforts to rebuild health systems in post-conflict contexts? And what impact do post-conflict interventions within the health sector have on gender equity within the health system? We focused our research on four contexts of post conflict health system engagement which spanned time and space: the Mozambican conflict ended in 1992, while post-conflict efforts started in Timor Leste in 1999/2000, and in Sierra Leone in 2002. In Northern Uganda, efforts to rebuild the health system began after mid-2006. These areas all experienced intense conflict which destroyed health infrastructure and interrupted the delivery of services, and received significant donor assistance to rebuild those systems.

To ensure that our methodological approach was rigorous, transparent and replicable, yet adaptable to the challenges of exploring a novel research area, the research team undertook a framework synthesis to investigate these research questions and present the research results. Framework syntheses enable a team of researchers to explore emerging research problems while maintaining the methodological standards of the systematic review. Framework syntheses begin with a policy priority or problem. Researchers borrow a tentative framework or develop that framework from key concepts that emerge in the research. The evidence base is then explored in a systematic, replicable manner, with the research team working to identify patterns in the data. The research team undertakes an iterative process of analyzing the available evidence, coding this research, and adjusting the framework as necessary. The team then draws conclusions from the evidence, outlines the limitations of the research, and works to identify further research that is needed [5].

Public health methods strive to ensure research results are unbiased, generalizable, and replicable. While the systematic review process is optimal to achieve these results, its methodology is designed for narrow research questions that investigate a specific health care intervention and depend on the pre-existence of an evidence base [5, 6]. Global health includes many critical policy challenges where peer-reviewed research is limited, available evidence is derived from various multi-disciplinary approaches not conducive to statistical comparison, and data is often not available or is of questionable quality [6]. Moreover, many global health policy issues are broad in scope, and relate to system level challenges rather than the efficacy of particular health interventions. To address such broad issues ore emerging research questions, like health interventions and gender equity, other valid approaches to systematic reviews have been developed, recognized and adopted [5, 7–9] such as the framework synthesis.

The research team selected the WHO Building Blocks as the framework to structure our analysis of gender equity in post-conflict health system engagement, recognizing the valid criticism of the technocratic nature of the building block approach [10]. Our review of the available evidence base was iterative in nature, taking place over the lifespan of our project (2012–2016). As the team moved forward with our research, we determined that the WHO building block framework needed to be adjusted to identify how to measure and assess gender equity in health systems. We therefore created gender equity benchmarks for each health system building block. Within each of these contexts, we examined peer-reviewed research, organizational reports, and literature from credible international health organizations and national reports from governments to assess if and how gender considerations were integrated into health system strengthening. As part of our effort to consolidate the findings of our project, in 2016–17, we undertook a structured review of the available evidence base, using a systematic approach utilizing set search terms and specific search engines. Table 1 shows the inclusion and exclusion criteria, as well as the evaluation criteria for the available evidence, and Table 2 shows the search strategy that identified this available evidence.

**Table 1:** Review Protocol

Research Questions How were gender considerations integrated within the efforts to rebuild health systems in the post-conflict contexts of Mozambique, Sierra Leone, Timor Leste and Northern Uganda? What impact did post-conflict interventions within the health sector have on gender equity with the health system?	
Inclusion Criteria	Exclusion Criteria
• Health interventions after peace agreement or cessation of hostilities (Mozambique after 1992, Timor Leste after 1999, Sierra Leone after 2002, Northern Uganda after 2006)	• Health interventions during active conflict
• Interventions that addressed one or all WHO Health System Building Blocks • Health interventions that addressed gender and access to health care (maternal services)	• Disease Specific Interventions, including HIV/AIDS
• Geographic scope: Research on Mozambique, Timor Leste, Sierra Leone, Northern Uganda	• Research from outside these four geographic areas
• English peer reviewed articles • English reports from UN Women • English reports from international non-governmental organizations • English reports from national governments	• Non-English peer reviewed articles • Non-English reports from governments
• Health system data from multilateral organizations • Health system data from national governments • Health systems data from Institute for Health Metrics and Evaluation	
Evaluation of Available Evidence • Examined available evidence to assess if and how health systems functioned as measured against the ‘benchmarks’ of gender equity; • Grouped evidence into themes under the benchmarks as common patterns appeared in the evidence.

**Table 2:** Search Strategy (2016–17)

Search Terms	Total Articles		Articles Reviewed	
“Mozambique AND. ..”	Promed	Scopus	Promed	Scopus
Health system	78	401	2	4
Health system gender	4		0	0
Health system access	42	94	1	5
Maternal health	191	176	1	8
Reproductive health	106	76	5	5
Health financing	34	46	1	0
Human resources	131	374	8	5
Health information	221	272	4	8
Community health workers	67	66	2	6
Gender mainstreaming	2	1	1	1
Health Governance	9	13	2	0
Gender Equity	4	13	3	3
Health Equity	26	23	8	2
“Timor Leste AND. ..”	Promed	Scopus	Promed	Scopus
Health system	24	58	2	2
Health system gender	4	0	0	0
Health system access	3	9	2	2
Maternal health	26	34	3	3
Reproductive health	10	13	2	3
Health financing	10	9	0	0
Human resources	15	54	1	2
Health information	32	39	1	1
Community health workers	11	10	1	1
Gender mainstreaming	0	2	0	0
Health Governance	2	4	0	0
Gender Equity	1	1	0	0
Health Equity	3	2	0	0
“Sierra Leone AND. .. ”	Promed	Scopus	Promed	Scopus
Health system	105	212	5	5
Health system gender	2	1	0	0
Health system access	12	38	2	5
Maternal health	73	97	3	7
Reproductive health	33	31	2	2
Health financing	12	14	0	2
Human resources	65	152	0	3
Health information	84	117	2	4
Community health workers	35	46	1	2
Gender mainstreaming	0	0	0	0
Health Governance	7	11	0	0
Gender Equity	0	3	0	0
Health Equity	18	7	0	0
“Northern Uganda AND. .. ”	Promed	Scopus	Promed	Scopus
Health system	37	43	3	3
Health system gender	2	2	0	2
Health system access	8	11	1	2
Maternal health	22	23	2	2
Reproductive health	24	21	4	4
Health financing	0	0	0	0
Human resources	11	40	1	2
Health information	38	48	1	2
Community health workers	19	16	1	1
Gender mainstreaming	0	2	0	0
Health Governance	0	0	0	0
Gender Equity	2	0	0	0
Health Equity	0	0	0	0

We supplemented the published studies gathered through our search of the evidence base with an analysis of health data. Data for all four contexts is poor, and sex-disaggregated data was not always available, nor was data disaggregated for sub-national regions (important for Northern Uganda). In the absence of functioning civil registration systems and health information systems, researchers and policy makers rely on Demographic Health Surveys (DHS) and regional household surveys that target specific diseases or issues. We recognize that the reliability and validity of such data can be questionable, [11–13] requiring triangulation from other sources which is seldom undertaken. Where possible, we therefore utilized health data from the Institute for Health Metrics and Evaluation (IHME), an organization that undertakes careful validity checks on the data it gathers and presents.

### The gender blind nature of health system interventions

If and how health systems interventions impact on gender equity is important for policy makers but challenging for researchers. Understanding the complex myriad of factors that contribute to health system improvements, or to health system deterioration, is very difficult. There is no agreement on how to undertake such an exercise – health systems frameworks describe the various components of health systems, but they do not explain or predict how interventions in the health sector will drive changes in health system outcomes or health status [14–16].

Moreover, the relationships among gender, politics, the economy, and the health system are deeply endogenous. How can analysts distinguish between a weak health system that lacks resources, and a health system that is gender inequitable [4]? The scope of gender inequalities, and specifically, violations of the rights of women and girls, has a profound impact on health outcomes [17, 18]. Disentangling the impact of these inequalities from inadequate provision of health services is challenging. Can analysts interpret positive or negative health outcomes as being a result of gender policies – or as a result of changes in the quality of health care services? Given the higher dependence of women on the health system as a result of their reproductive role, more data is available on the health of women. In the absence of data on the health of men and boys and the ability of the health system to respond to their health needs, are we making unfounded assumptions that health systems are gender inequitable and failing to deliver only for women and girls?

Such questions are difficult to answer. The absence of theories or frameworks that provide explanatory or predictive power make individual analysis of health systems challenging, and health system comparisons particularly difficult. Health systems literature and models provide little guidance on how to compare systems and which countries to select for that process [19, 20]. Yet comparative cases provide analytical richness, particularly in the early stages of research, and such comparisons can be critical for developing conceptual themes and frameworks. Moreover, without analysis across various contexts, health systems researchers would have little basis to demonstrate the generalizability of their findings beyond one country – which is of little use to policy makers working to strengthen health systems in multiple settings.

**Fig. 2:**
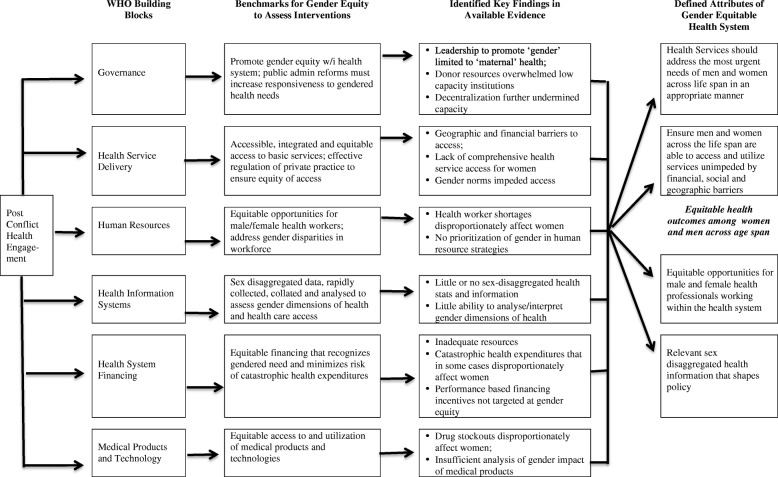
Development of Conceptual Framework for Study

To navigate these challenges in our research, we structured our analysis on the World Health Organization’s (WHO) six health system building blocks, which include governance, health service delivery, health information, human resources, financing, and medical products and technologies. We then applied a ‘benchmark’ approach. Benchmarking compares health systems against an agreed-upon standard or realistic goal of health system performance, and assesses if the health system meets that standard [21, 22]. The World Health Organization’s *World Health Report 2000* was one of the first and most prominent examples of the use of benchmarking in the analysis and the comparison of health systems against a set of objectives, although the report was devoid of gender analysis [19]. In our review, we developed benchmarks for gender equity in each building block that described how a ‘gender equitable health system’ would perform. Our review of the available evidence base in each context then assessed progress against these benchmarks. These benchmarks are outlined in Fig. 2. We found common themes in the four contexts, outlined in Fig. 2 and described in detail below. These key findings led us to define the attributes of a gender equitable health system to guide future research and policy.

### Introduction to the four contexts

The intensity and duration of the conflict varied across the four contexts. Mozambique and Timor Leste both experienced continuous strife after the end of Portuguese colonialism in 1974. Mozambique erupted into a brutal civil war from 1977 until 1992 that killed an estimated 1 million people. Over 100,000 people died during Indonesia’s occupation of Timor Leste and the brief war that followed its independence referendum in 1999 [23]. Sierra Leone’s decade long war, which killed an estimated 50,000 people and displaced an estimated 2 million, ended in 2002 [24]. Tens of thousands of people died during the twenty year long war in Northern Uganda, which ended in 2006 and displaced an estimated 1.9 million [25]. Although the Juba peace process failed (except for the cessation of hostilities agreement), the talks ushered in relative peace in Northern Uganda [26]. In all four contexts, more men and boys died in combat, while women and girls faced a higher risk and prevalence of sexual and gender-based violence [27–29].

While the post-conflict context is challenging for both women and men, conflict exacerbates gender inequalities as well as inequitable health outcomes. Throughout all four settings, the disruption to health services that accompanied the conflict disproportionately impacted women and girls because of their reproductive and caregiving roles [26, 30, 31]. Our research shows that the pernicious interaction between gender inequality and health outcomes lingered in the post conflict period. In Northern Uganda, the Gender Development Index remained lowest in areas previously affected by the conflict. Many women experienced a significant decline in income from 2009 to 2013, and gender-related inequalities remained entrenched [26]. IDPs returned to their home villages where few or no health services were available [32]. While Mozambique has been praised for its strong political commitment to gender equality, health indicators for women and girls show pervasive discrimination: between 1990 and 2010, women aged 25–29 experienced the largest increase in mortality with a 207% increase [33], maternal mortality remained stubbornly high and women and girls were disproportionately affected by HIV and AIDS [34].

In Sierra Leone, customary law reflects and reinforces cultural norms, granting men rights over the land and resources and sanctioning violence within marriage [35, 36]. As a result, our findings indicated that female genital mutilation and domestic violence remain widespread [37]. Similar customs limited women’s access to land in Northern Ugandan [38]. In Timor Leste, social norms that devalue women remained pervasive. Violence against women was widely accepted in intimate relationships [39]; UNICEF reported that over 80% of men, and 86% of women believed that wife beating is justified [40]. Health indicators for women remained problematic, and in the immediate post conflict period, women experienced one of the highest rates of maternal mortality in the world (953 per 100,000 live births). Maternal mortality declined in the post-conflict period, but remained more than five times the regional average and double the average for developing countries [41].

Despite rhetorical efforts to promote gender in the post-conflict period, gender indicators remained disappointing across all four contexts, as is clear in Table 3. Women’s life expectancy was higher than men, in part due to biology as well as male engagement in higher risk behaviours, including occupational hazards and their role as combatants. Maternal mortality rates, fertility rates, contraceptive prevalence, and HIV/AIDS prevalence rates reflected gender inequities. Literacy rates, GDP per capita, and labour force participation rates revealed that women remain disadvantaged in these areas across the four contexts and illustrate pervasive gender inequalities throughout society.

**Table 3:** Population, Economic Indicators, Social Development Statistics and Health Indicators

	Mozambique		Timor Leste		Sierra Leone		Northern Uganda^a^	
	Women	Men	Women	Men	Women	Men	Women	Men
Population Indicators								
Population	28 million (2015)^b^		1.13 million (2013)^c^		5.98 million (2012)^d^		14.46 million^e^	
Life Expectancy	59.9^b^	54.3^b^	70.1^f^	66.5^f^	46^d^	45^d^	57.6 (2011)^e^	52.9 (2011)e
Percent of Population under 15	(2012) 45%^g^		46% (2013)^c^		41.74% (2012)^d^		Not disaggregated for Northern Uganda	
Economic Indicators								
Development Assistance for Health 2014 (million USD)^h^	864.54		22.78		179.84		Not disaggregated for Northern Uganda	
Estimated Gross Domestic Product per Capita 2014 (2011 PPP $)^i^	1040	1210	3122	7530	1582	1981	Not disaggregated for Northern Uganda	
Social Development								
Some Secondary School Education (percent)	1.5^j^	6.0^j^	No data available		No data available		6.3 (2012)^e^	26.4 (2012)^e^
Literacy % of total population	28 (USAID 2016)	60 (USAID 2016)	43 (UNDP 2011)	59 (UNDP 2011)	32.6^k^	54.7^k^	53.2%^e^	N/A
Labour Force Participation Rate (percent)	26.3^j^	75.8^j^	24.6^f^	50.8^f^	65.7^l^	69^l^	88.9 (2012/13)^e^	90 (2012/13)^e^
Selected Health Statistics								
Fertility Rates	5.9 (2011)^m^	N/A	5.9 (2013)^c^	N/A	5 (2012)^d^	N/A	6.8 (2011)	N/A
Contraceptive Prevalence (% women aged 15–49)	12^n^		22.3^o^	N/A	11^p^	N/A	Not disaggregated	N/A
Antenatal care % at least 1 visit	90.6^q^	N/A	84.4^o^	N/A	93^r^	N/A	Not disaggregated for Northern Uganda	
Skilled attendant delivery %	54.3^q^	N/A	61^p^	N/A	62.5^r^	N/A	Not disaggregated for Northern Uganda	
Maternal Mortality Ratio	248.7 (151.4–365.4)^s^ 490^j^	N/A	270^c^	N/A	1100 (2013)^d^	N/A	438 (Uganda)^e^	N/A
HIV Prevalence – 15-49 years	10.8%^t^		Not reported^o^		1.5%		Not disaggregated for Northern Uganda	
HIV – Children 0–14 years^t^	190,000 (est)		Not reported^o^		5800		Not disaggregated for Northern Uganda	
HIV Prevalence – Young adults (15–24)	11.1% (est)^t^	3.7% (est)^t^	Not reported^o^		1.0	0.3	Not disaggregated for Northern Uganda	
HIV – Adults 15–49	1,400,000 (est)^u^		Not reported^o^		No data available		Not disaggregated for Northern Uganda	
	820,000 (est)	580,000 (est)						

^a^Very few figures are available that are disaggregated for Northern Uganda – much of the data is available only at the country-wide level

^b^Mozambique Country Profile. Institute for Health Metrics and Evaluation; 2017. http://www.healthdata.org/mozambique. Accessed June 12

^c^WHO. Timor-Leste: WHO Statistical Profile. Dili: WHO; 2015

^d^WHO. Factsheets of Health Statistics. Brazzaville: WHO Regional Office for Africa; 2014

^e^UNDP. *Uganda Human Development Report 2015: Unlocking the Development Potential of Northern Uganda.* Kampala: UNDP;2015

^f^UNDP. *Timor-Leste: Briefing note for countries on the 2015 Human Development Report.* New York: United Nations Development Programme;2015

^g^WHO. Mozambique: Country Cooperation Strategy at a glance. Maputo: World Health Organization; 2014

^h^IHME. Financing Global Health 2016: Development Assistance, PUblic and Private Health Spending for the Pursuit of Universal Health Coverage. Seattle: University of Washington; 2017

^i^UNDP. 2015 Human Development Statistical Tables: Gender Development Index. New York: UNDP; 2015

^j^UNDP. Human Development Report 2014: Explanatory Note on the 2014 Human Development Report composite indices Mozambique. *Sustaining Human Progess: Reducing Vulnerabilities and Building Resilience*. New York: United Nations Development Program; 2014

^k^Mundi i. Sierra Leone Literacy. 2011; http://www.indexmundi.com/sierra_leone/literacy.html. Accessed May 13, 2016

^l^UN Data. Sierra Leone. New York: United Nations; 2013

^m^PRB. Fecundidade e Planeamento Familiar no Inquérito Demográfico e de Saúde de Moçambique 2011 (IDS). In: Estatistica INd, ed. Maputo: Insituto Nacional de Estatistica & Population Reference Bureau; 2013

^n^WHO. *Mozambique: health profile.* Geneva: World Health Organization;2012

^o^ UNICEF. At a glance: Timor-Leste. 2013. Accessed January 25, 2016

^p^WHO. Sierra Leone: Factsheet of Health Statistics. Brazzaville: WHO Regional Office for Africa; 2014

^q^UNICEF. Information by Country: Mozambique Statistics. 2012; http://www.unicef.org/infobycountry/mozambique_statistics.html. Accessed August 21 2012

^r^UNICEF. At a glance: Sierra Leone. 2013; http://www.unicef.org/infobycountry/sierraleone_statistics.html. Accessed May 13, 2016

^s^Nicholas Kassebaum et al. Global, regional, and national levels and causes of maternal mortality during 1990–2013: a systematic analysis for the Global Burden of Disease Study. *The Lancet.* 2014;384:980–1004

^t^CNCS. Global AIDS Response Progress Report. Maputo: Conselho Nacional de Combate ao HIV e SIDA 2014

^u^UNAIDS. Mozambique: HIV and AIDS estimates. Geneva: UNAIDS; 2013

### Post-conflict health system engagement and gender

In the post-conflict period, governments and donors often initiate full or partial health system reforms in an effort to make the system more effective and efficient. Initiatives include efforts to improve the delivery of health services, reform human resources and restructure the distribution of tasks among health workers, and implement new finance mechanisms or payment modalities. Such reform efforts create a path dependency: once structural changes are implemented, efforts to introduce subsequent reforms that diverge from the existing system are extremely difficult to implement. Therefore, health system engagement in the immediate post-war period can be critical to initiating the health system on a path towards gender equity.

While all four contexts saw the rebuilding of health services and infrastructure, the extent to which governments and donors engaged in health system reform differed. While Mozambique launched the *Health Sector Reform Programme*, the government maintained the pre-conflict system built after the country achieved its independence in 1975. Post-conflict health interventions repaired the damage from war, but did not address fragmentation in delivery or insufficient and inefficient health care financing [42, 43]. In Northern Uganda, as part of their broader development plan in the post-conflict period, the government committed to the integration of the north within the broader Ugandan health system, including efforts to broaden access to health care services in the north [44–46].

Timor Leste not only rebuilt the health infrastructure destroyed by conflict, but also worked to reform the system into a more efficient, primary care based system [47, 48]. The Sierra Leonean government also undertook significant reform, by introducing a national decentralization policy that included the health sector [49] and a “Free Health Care Initiative” (FHCI) to provide free health services to pregnant women, lactating mothers and children under five. However, decentralization was plagued with systemic failures, clearly evident during the 2014–6 Ebola Virus Disease (EVD) outbreak with local responses undermined by failures at the national level [50–52]. The Ebola outbreak further weakened Sierra Leone’s health system, with maternal health services heavily affected. Post-Ebola health system measures have been criticized for their failure to integrate gender equity as an explicit objective [52, 53].

### Applying the benchmarks: Assessing gender equity in the health system

Within each WHO building block, we developed clear benchmarks to evaluate if health systems were gender equitable, and to assess if health system interventions improved gender equity in the post conflict period. To strengthen our analysis, we attempted to distinguish between those results that reflected a weak health system, from those that specifically reflected gender inequity.

#### Building block one: Health systems governance benchmark

Our benchmark for gender equity in health systems governance is the following: The government must meaningfully promote gender equity within the health system, while public administration reforms in the health sector, such as decentralization of responsibility for health care to provincial and district levels, should increase the responsiveness of the health sector to the differential needs of men and women [4]. In our cases studies, we assessed this benchmark by examining leadership practices within the health sector, donor engagement to promote more effective health care administration, and decentralization of authority for health services to the local level.

##### Leadership

Two of the four contexts made efforts to promote gender issues within the health system. Mozambique committed to the importance of gender equity in the health sector early in the post-conflict period. It quickly established Gender Focal Points within the Ministry of Health and developed strategies for integrating gender within health policy. Yet Gender Focal Points faced several obstacles:

The limited expertise in gender mainstreaming skills, the lack of capacity in gender sensitive policy formulation and programme analysis, the feminization of certain conditions such as STI/HIV/AIDS and the poor integration of initiatives against gender-based violence are some of the challenges to overcome ([54] p. 6).

Moreover, what the Ministry of Health considered as “gender” was confined to interventions on ‘maternal conditions’, sexual violence, and HIV/AIDS – particularly Prevention of Mother to Child Transmission (PMTCT) [55]. This finding is consistent with evidence from other contexts – gender mainstreaming is generally interpreted as ensuring health systems provide maternal health services, not necessarily ensuring that they respond to the differential health needs of women (and men) across the lifespan.

Timor Leste also incorporated gender equity within its 2002 National Development Plan. It mainstreamed gender health concerns in all programmes, worked with relevant sectors/organizations to advocate for improved status for women, and promoted equal rights for men and women [56]. However, overworked and understaffed Ministry of Health officials struggled to consistently integrate gender considerations into their work [57]. Tellingly, the 142 page National Health Sector Strategic Plan for 2011–2030 does not include the word ‘gender’ [58]. Therefore, the degree to which gender interventions were meaningfully promoted is questionable.

##### Donor influx and fragmentation

All of the contexts experienced an influx of donor resources in the immediate post-conflict period. The degree to which this donor involvement facilitated more gender equitable health systems is not clear. Donors channelled funds and attention to maternal health and sexual violence [1, 59], but donor demands also strained the systems’ capacity in the different contexts. In Timor Leste, the Minister of Health “felt that due to severe staff constraints, the measures included in the [reform proposals] were driven by consultants and donors, with insufficient consultation with Timorese,” ([60] p. 29) a sentiment echoed by others [61]. Mozambique encountered similar problems [43]. To circumvent such fragmentation, in 2001 Mozambique adopted a Sector Wide Approach (SWAP) guided by Health Sector Strategies and promoted donor use of a common fund (PROSAUDE) to ensure that donor efforts in the health sector were better coordinated and focused on strengthening the health system [42, 43, 62]. Despite these efforts, the Mozambican Ministry of Health had insufficient capacity to both manage the demands of various donors and effectively govern the health system [63].

##### Decentralization

Responsibility for governing health services is often decentralized to the local level, in hopes that this will enhance the responsiveness of the health system. A more responsive health system is better able to address the particular health needs of women, men, girls and boys, and should in theory be more gender equitable. However, if decentralization is undertaken without the requisite human, institutional and financial resources, and if central departments are reluctant to cede their authority [4], the responsiveness of the health system at the local level will be undermined, including its ability to respond to gendered health needs.

Both Uganda and Mozambique undertook decentralisation in the 1990s, giving districts the responsibility to deliver health care and determine the priority health needs of the population [42]. Timor Leste and Sierra Leone followed in the early 2000s [64, 65]. In Northern Uganda, donor engagement and demands for accountability caused a recentralization of priority setting [66]. In Mozambique “it is not clear which activities are within the scope of control of district, provincial or national managers” ([67] p. 9). In Sierra Leone, this lack of authority at the local level was clear during the Ebola outbreak: districts waited for guidance from the central Ministry instead of acting quickly [51]. In all four contexts, the districts’ budget priorities were constrained by central government decisions. While on paper, districts had greater power, they had insufficient funds (and sometimes authority or decision-making space) to fully implement programs that better reflected local realities.

To summarize the findings for gender equity within this ‘governance’ building block, the review found that donors, international and national stakeholders paid lip service to gender. Donors did not address the differential vulnerabilities of women, girls, boys and men, nor did they apply gender analyses to their health system engagement. They tended to focus on maternal health and did not allocate sufficient resources to ‘mainstream’ gender during the rebuilding of the health system. Moreover, the influx of donor resources as well as efforts to decentralize worked to undermine the capacity of national stakeholders to implement health system interventions – including the minimal efforts to ‘mainstream gender’. The inability of health systems to effectively manage donor engagement as well as the decentralization process reflects their fundamental weakness, but also impacted negatively on gender equity given women and girls' higher reliance on health services.

#### Building block two: Health service delivery

Our gender equitable benchmark for health services is the following: To be gender equitable, health services should ideally be accessible, integrated to ensure an efficient provision of a basic or essential package of health services, and effectively regulate private practice. Below we analyse access to health services (with a particular focus on geographical, financial and cultural barriers) as well as if and how the integration and delivery of health services has impacted on gender equity. In our review, we found little information on regulating private practice, with much of the research focused on barriers to health services access.

In all four contexts, governments committed to a minimum package of health services, particularly for pregnant women and children. The Timorese Constitution recognized the right of every citizen to health and medical care and obligated the State to establish a universal and free national health service [56]. Sierra Leone adopted the Free Health Care Initiative (FHCI) to ensure women and children have access to services, which initially led to an increased use of services among pregnant women [68] and children under five [69]. Uganda committed to providing a National Minimum Health Care Package [70, 71], and Mozambique also adopted a basic package of services for maternal, neonatal and child health, delivered through the community by local health posts.

This commitment to health services increased utilization, for example, in Sierra Leone 45% more pregnant women made one antenatal care visit and ‘new acceptors’ of family planning increased by 140% [68]. Yet across all four contexts, the quantity and quality of health services remained insufficient, focused on maternal health services, with significant geographic, financial, and cultural barriers undermining access to the services that exist. Moreover, none of the interventions worked to promote gender equity within the system.

##### Geographic and financial barriers to access

Available evidence from our four contexts focused primarily on rural access and underscored the scarcity of health services in rural areas and how this disproportionately impacts on women. In urban areas, some research suggested that residents will bypass nearer facilities for higher quality or lower cost health clinics that are further away [72]. In rural areas residents had few health care options available.

In Timor Leste, there are only six district referral hospitals, 67 community health centres, and 213 health posts. To serve locations that lack health facilities, Timor Leste implemented Servisu Integradu da Saude Comunitaria (SISCa), a community-based system of health service delivery that includes treatment of infectious diseases, improving nutrition, maternal and child health, and family planning [64, 73]. Even with these reforms, health care remained insufficient [41]. Approximately 20 to 30% of the population still lacked access to services [64]. Women in rural areas were much less likely to have access to health services than those in urban areas [74, 75], largely due to the constraints on infrastructure, which included impassable roads, lack of transportation to health facilities, unreliable supply chains, lack of electricity and lack of training opportunities for health workers [41, 76, 77]. Women who lived within 5 km of a health centre were more likely to deliver at a health facility, with facility based births declining with increased distance from the health centre [78]. In one rural area alone, only 50 births out of 2000 were carried out in the district hospital [76].

Mozambique also faced a shortage of health clinics, and fell below the ratio of 10,000 inhabitants per primary care unit [79]. Geographic distance and the lack of transportation to health facilities were significant barriers to antenatal care in rural areas [80] as well as access to family planning [81], with many Mozambicans walking more than an hour to access health care [82]. Geographic barriers significantly worsened during the wet season [83]. The lack of transportation added another barrier to timely access to health services [84]. Geographic barriers also impacted on awareness of health care issues; women who lived further away from health facilities were less likely to be aware of the importance of voluntary counseling and testing for HIV/AIDS [85].

In Sierra Leone, geographic barriers to health service access remained significant, and were exacerbated with the impact of Ebola on the health system [86]. Rural women were particularly hard hit; challenges included difficult terrain which affected access to health services, and inadequate numbers of skilled birth attendants. “Regional and socioeconomic disparities in women’s and girl’s access to sexual and reproductive health services, including skilled birth attendance and adequate antenatal and postnatal care, affect mainly rural women, women in Northern Province, poor women and women with low levels of education” [87]. Long distances and lack of transport to health facilities were also barriers to health care access in Northern Uganda [32].

Financial barriers also existed across the four contexts, despite the policy of providing essential services for free. Health workers often requested personal payment from patients. A study in Northern Uganda found that over half of respondents reported that health providers extorted money: “When I went to give birth, the nurse told me that ‘since you have given birth well I want you to give me something but don’t tell the in-charge (supervisor)’. Then I removed 5,000 Shillings and gave her” ([88], p. 8). In Sierra Leone, researchers found that in some cases patients were asked to pay for the free cards that recorded antenatal and child vaccinations [89], and patients in Mozambique reported extra payment for ‘free’ services [90]. This financial burden falls disproportionately on women, given their greater reliance on health services and their caregiving roles.

Other financial barriers included the cost of researching those services, as well as the need to purchase certain supplies and food once they are in the hospital [90–92]. As a result of their inability to afford these supplies, and the accompanying shame, some women avoided facility delivery [32, 92]. In Timor Leste, lower income women were less likely to utilize a health care facility for childbirth [74]. When women gave birth at health clinics far from home, they also faced more time away from paid work, and lost wages presented another barrier to health care access [91, 92]. Across these four contexts, research showed that women are reluctant to leave their income earning opportunities [74]. Women who are single, or whose partners do not share or assume the cost of medical expenses are particularly vulnerable [90].

##### Lack of access to satisfactory and comprehensive health services

Across the four contexts, the quality of health services was inadequate, and individuals lacked access to satisfactory and comprehensive services. Again, due to women’s reproductive and caregiving roles, they were particularly hard hit. In all four contexts, women and girls faced challenges accessing comprehensive sexual and reproductive health services [66, 75, 80, 87, 91, 93–95].

In Sierra Leone, an inadequate and demotivated health work force, few financial and non-financial incentives for health workers, and inadequate infrastructure and equipment undermined comprehensive access to health care [96]. Given the dependency of women on the health sector, this particularly impacted on their health outcomes, clearly shown by antenatal care and maternal mortality rates in Table 3. Across all four contexts, health worker absenteeism was widespread [82, 97]. In Mozambique, facilities are often poorly stocked, lacking electricity (55%) and running water (41%) [82]. A 2009 study showed that poor infrastructure as well as shortages of drugs and medical products undermined health services for women, men, girls and boys [97]. Vertical delivery silos, caused by separately funded programs, required patients to obtain referrals from one service to another, including from Maternal and Child Health to HIV care [80, 98], which brought increased financial costs and opportunity costs which women find particularly challenging. In Northern Uganda, respondents in one study cited government health clinic’s late opening hours (10 am) and early closure (1 pm) as an impediment [99], a problem also faced by patients in Timor Leste [41].

While insufficient research has been conducted on individuals with disabilities, evidence from Sierra Leone suggests that people with disabilities are less able to access public health services, although women with disabilities reported no significant difference in access maternal health care [100].

##### Gender and cultural norms that impede access

Health system research often focuses exclusively on geographic and financial barriers but neglects analysis of how gender and cultural norms influence the ability of individuals to access health care. The lack of respect from health workers, and their abusive and degrading behaviour toward women, emerged as an important factor that undermined their willingness to seek care across all four contexts [32, 77, 84, 99]. This abusive behaviour reflected gender norms across all four settings that devalue women and girls. This treatment of women and girls and its impact on access provides evidence that health systems are clearly gender inequitable – not simply lacking financial resources.

One main barrier identified across the sites, and especially in Northern Uganda, included past unpleasant experiences or fear of such experiences at the hands of health providers at the health facility, discouraging some women from seeking services (60%). With extensive impoverishment among the rural women who were temporarily displaced from their communities during the conflict, many of them felt despised, looked down upon, and poorly received by health personnel when visiting the health facility ([88] p. 6).

Research in Mozambique also cited the “unfriendly environment” in health centres, including health workers’ disrespectful treatment of women during childbirth [84, 101], despite the government’s humanizing initiative [80]. Female sex workers acutely felt such barriers, as they did not trust health workers to treat them with respect, compassion or to maintain privacy and confidentiality [102]. Patients in Timor Leste also reported disrespectful behaviour that included shouting at patients, blame and shame attitudes as well as nepotism [41]. Ensuring appropriate care “did not require complex or expensive technology, a major overhaul or significant costs. Rather, it implied listening to and observing the experience and feeling of women patients, encouraging and supporting staff to embrace change, and monitoring the process and data to observe the impact” ([32] p. 11).

Cultural norms emerged as another important factor impeding access to health services. In Northern Uganda, several studies found that women did not attend health facilities for antenatal and delivery care because “People think that when you are pregnant it is a normal condition and you do not have to go to the health facility. They feel that when you go there you are a coward” ([88], p. 8). Such cultural norms underscored the importance of health workers developing a plan for delivery at a health facility [32, 41, 74, 77, 103]. In Sierra Leone, childbirth is often accompanied with a set of rituals, particularly in rural areas, which can only be provided at home [104, 105]. While in parts of Mozambique, “fears of witchcraft and bad spirits were so strong that many women hid their pregnancies and delayed going to the maternity clinic” ([106] p. 366).

In addition, our research showed that across all cases, women were reluctant to leave their homes, in large part due to their gendered household responsibilities [84, 88, 92]. Women also often lacked the ability to autonomously decide to deliver in a health centre. Researchers underscored that health care decisions are complex [92], socially negotiated, and are rarely made by women alone, but are “an amalgamation of the many people involved” in the woman’s life ([104] p. 8]. A study in Northern Uganda found:

“One-quarter of women indicated they would decide for themselves where to deliver, while 32% said their husband or partner would decide, 30% said the decision would be taken jointly between themselves and their partner, and 11% indicated others would decide – including mother, mother-in-law, other family member or traditional birth attendant” ([32] p. 6).

Women may also be prevented from leaving the house to seek care. In Maputo, Mozambique in 2011, skilled health workers attended only 54% of live births [107]. “Reportedly the decision to seek care was taken by the woman herself in [only] 29.3 percent of the near miss cases, while in the remaining cases, the woman depended on the husband’s or other family members’ decisions” ([108] p. 4).

Pernicious gender norms deeply rooted in cultural beliefs also affected access. In both Mozambique and Sierra Leone, research found that women who experienced protracted labour feared being accused of infidelity. Tradition dictates that if a woman has a long childbirth, it is a sign that the child is illegitimate. In some cases, the family interrogates the woman on her suspected infidelity, which delays access to health services [109]. In Sierra Leone, researchers found:

“ [. . .] another reason women did not reveal the onset of their labour related to the fear of being accused of immoral behaviour if the labour was considered to be taking too long. Many participants in the villages spoke about infidelity as a potential cause for a delayed or obstructed birth as another man’s sperm had ‘contaminated’ the pregnancy. In such cases the woman in labour needed to ‘ speak out’ or confess to the adultery in order for the birth to proceed” ([104] p. 7).

For women living with HIV/AIDS, stigma and fear undermine access to health care. “For women, disclosing their health status could lead to divorce, which would result in the loss of their children and their financial stability, as men could force them to leave the family home” ([110] p. 7).

To summarize the findings for gender equity in the building block of ‘health service delivery,’ the review found that women lacked equitable access to health services for a number of reasons, specifically geographic and financial barriers, as well as fear of abusive behaviour from health care staff, and specific cultural and gender norms surrounding reproductive health, sexuality and childbirth. While some of these barriers – namely geographic and financial – affected all those who utilize health services, women were disproportionally impacted due to their reproductive and caregiving roles and as a result of pernicious gender norms. Health system interventions did not work to address these differential vulnerabilities, nor did they integrate gender analysis to their interventions – they instead seemed blind to the pernicious impact of gender inequality on health outcomes.

#### Building block three: Human resources

Our gender equitable benchmark related to human resources is the following: Gender equitable systems promote equitable opportunities for both male and female health workers across all cadres and ensure that gender disparities are addressed in health worker advancement, planning, retention, supervision and remuneration across all areas of the health workforce. Below, we assess this benchmark by examining health worker shortages and human resource practices.

##### Gendered impact of health worker shortages

Severe shortages of health workers characterized all four contexts. Mozambique had 0.03 physicians per 100,000 population – one of the lowest rates in Sub Saharan Africa [107]. Sierra Leone was in a similar situation with only 0.02 physicians per 100,000 population [111], and some of these physicians died during the EVD outbreak. Timor Leste also faced serious human resource challenges [103]. Compounding the shortage was the participation of many health workers in largely unregulated private practice. In Mozambique, by 10 am, many doctors had left the public hospitals for private facilities [112]. Research shows that female dominated health professions, such as nurses and midwives, were affected by this shortage of doctors [113], facing a heavier workload and additional responsibilities. In Mozambique, nurses were in short supply, at 3.4 per 10,000 [114], while midwives were also extremely over-stretched [101]: Mozambique needed to “double its number of midwives to remedy shortages, in addition to strengthening skills of the existing workforce” [97].

##### Prioritization of gender in human resource planning

We found little evidence that gender was prioritized in recruitment and promotion, or in broader human resource strategies. Mozambique had multiple training programs in place to augment resources for health, and while the university medical school was committed to training more female doctors, we found no evidence that the Ministry prioritized gender equity in its overall human resource strategy [115, 116]. The promotion of women was also not part of the recruitment or planning process for Community Health Worker Program (Agente Polivalente Elemantar or APE) [117]. While there was evidence of an increasing number of female physicians within Maputo [118], the majority of community health workers in some regions – particularly in the North of Mozambique - were men [119]. Gendered barriers also undermined female participation and advancement. In Sierra Leone, female health workers in rural postings often had to be separated from their families, and faced personal security challenges due to the lack of provision of transportation to travel to and from the health centre [96]. In addition, female community health workers were often unpaid, reflecting the lack of value placed on the economic contribution of women, furthering their dependence on family members (often male) who were financially remunerated, which undermined their ability to escape poverty [120]. Community health workers also had to navigate barriers – such as a lack of education and restricted mobility – that are rooted in gender norms that devalue women and undermine their freedoms [121].

To summarize the findings, our review found severe shortages of health workers impacted on everyone, but disproportionately affected women given their reliance on health care services. In addition, female dominated professions, such as midwives and nurses, faced additional pressures with physician shortages, including when physicians leave the public system for their private practices. Moreover, we could find no evidence that gender considerations were integrated into human resource planning within these four contexts.

#### Building block four: Health information systems

Our benchmark related to gender equity in health information systems is the following: health information systems should identify gendered dimensions of health outcomes. Data should be sex-disaggregated, and health systems should ensure the rapid collection, collation and analysis of that data.

While health information systems were clearly weak across all four contexts, evidence on efforts to strengthen these systems and the analysis of health data was scarce. In Timor Leste, the Ministry of Health worked to provide sex-disaggregated health statistics into its Health Management Information System [73]. Record keeping and data management was an ongoing challenge in Sierra Leone, and the National Health Sector Strategic Plan (NHSSP) included a table of performance indicators, but these were not sex-disaggregated [122]. In Mozambique, the development of the health information system was a stated priority, but progress remained slow. In many rural areas, health information remained in paper form and individuals did not have their own clinical record – their health profile and history was recorded in a central registry [97]. At the central level, the Ministry of Health lacked the capacity to fully analyse the data they received and present their findings to policy makers. The lack of analytical capacity was exacerbated at the district level [67].

To summarize, the review found very little research on gender equity within the ‘health information systems’ building block. Health data was clearly scarce. Without valid and reliable sex-disaggregated data that can provide insight into the health issues faced by men and women, and the different manner in which men and women engage with and are served by the health system, it is challenging for policy interventions to determine how to build a gender equitable health system.

#### Building block five: Health system financing

Our benchmark related to gender equity health financing is the following: the allocation of financial resources should be transparent and in a manner that reflects the gendered dimensions of health. Financing systems must be equitable, minimizing the risk of catastrophic health expenditures [4]. When evaluating health systems against this benchmark, it is challenging to differentiate between health systems that are gender inequitable versus health systems that are under-resourced. Under-resourced systems disproportionately affect women. Given that women have less disposable income (as clear in Table 3) and also utilize health services more, the failure of health financing systems to provide universal access to essential health care services has a particularly devastating impact on them. Below, we analyse the gendered impact of inadequate resources and catastrophic health expenditures, as well as the impact of financial performance incentives.

##### Inadequate resources, reliance on donors

Despite increased donor resources for health services, financial shortages undermined the equitable provision of health services across all four contexts. In 2015, Mozambique spent 9% of its total budget on health [123], while Sierra Leone spent 11.4% in 2013. Timor Leste government expenditure was even lower at only 3% in 2013 [124]. There were no disaggregated figures available for Northern Uganda, but the country as a whole spent 7.2% of its budget on health [125].

As a result of limited domestic resources, all four contexts relied heavily on external donors to fund the provision of health services. While this infusion of resources is essential, it makes countries vulnerable to donors, their priorities, and budgeting cycles. While Mozambique has a publicly funded health system, of the total amount spent in the health sector in 2014, 45% was from external donors (which included off-budget and vertical projects) [123]. In 2013, Mozambique received a total of 672.83 million USD in development assistance for health [126]. In contrast, Sierra Leone, with similarly challenging health indicators, received comparatively little prior to the Ebola crisis, only 57.22 million USD in 2013 [126] which increased to 322 million USD in humanitarian funding after the Ebola outbreak in 2014, most of which was directed towards health related activities [127]. The degree to which these funds succeeded in strengthening the health system, rather than simply addressing the immediate needs of the Ebola outbreak remain unclear.

##### Catastrophic health expenditures

Across the four contexts, patients often purchased private care, or made out of pocket payments to providers in public facilities to guarantee service provision. In Mozambique, catastrophic expenditures contributed 22.3% to poverty deprivation [128], and out of pocket payments accounted for 58.3% of total expenditure for health [129]. Uganda abolished user fees in 2001, but the perceived poor quality of services in public health facilities and long queues boosted demand for private health care. Given their greater need for health services, financial barriers to health services disproportionately affected women. Analysis from Northern Uganda highlighted the challenges faced by poorer older women (widows) to access quality care in the post-conflict period [130].

##### Financial incentives for health system improvement

Over time, donors experimented with financial incentives to improve the effectiveness of the health system. Such incentives are being used to improve the efficiency of the pharmaceutical and medical supply in Mozambique [131]. In Sierra Leone and Northern Uganda, donors initiated performance based financing to improve the overall effectiveness of both primary and secondary health care services [132, 133]. For Sierra Leone, these incentives targeted child and maternal health. While such funding encouraged higher performing health systems and directed resources to maternal health, researchers have not analysed their impact on gender equity. In Northern Uganda, not-for-profit providers were perceived as more efficient and participated in some donor-supported pay for performance initiatives [132]. While these institutes, mostly religiously based, provided health care of good quality, they did not always ensure comprehensive access to sexual and reproductive health services [134]. Donors appeared reluctant to address gender through performance based financing projects, as they were concerned that gender equality is perceived as a ‘western imposed’ agenda [135, 136].

To summarize the findings, our review found (unsurprisingly) that health services were inadequately funded across all four contexts. This undermined the quality of health care as well as the ability of health services to respond to differential needs of men and women. We found no evidence that health financing programs integrated an analysis of the differential vulnerabilities of men and women. Gender equity was simply not a goal of health financing strategies.

#### Building block six: Medical products and technology

Our benchmark for gender equity related to medical products and technology is that health systems should ensure equitable access to and utilization of medical products and technologies. To assess this benchmark, we examined the gendered impact of drug stockouts and pharmaceutical policies within our four contexts.

##### Drug Stockouts

Frequent drug stockouts caused by poorly functioning supply chains characterized all four contexts. Given their reproductive and caregiving roles, women were disproportionately affected by these shortages. Antenatal care and pregnancy interventions are drug-intensive, yet in one Mozambican study, the 42 medicines, commodities or lab tests required for appropriate antenatal and maternal care were not available at any one time in the studied facilities [80] A nurse observed:

“We do not measure the blood pressure because we do not have the device. We have many tensiometers but we do not have the stethoscopes. We only received sphygmomanometer [ . . . ] I’m here since February, and we haven’t assessed pregnant women’s blood pressure [. . .]” ([80] p. 5).

Another study in Mozambique found that shortages of oral and injectable contraceptives meant that women sometimes had to change their method of birth control [137]. In Timor Leste, a study found “[.. .] there were persistent and long term stockouts of essential medicines, sometimes for months at a time, including for the most basic items such as antibiotics, paracetamol, ibuprofen, iron supplements and oxygen for the clinic and patient transfer vehicles” ([41] p. 10).

In Sierra Leone and Northern Uganda, patients encountered frequent shortages of pharmaceuticals, and patients were often forced to pay for medications [89, 138]. Moreover, in Northern Uganda the extent of health provision by faith-based organizations undermined contraceptive prevalence:

Only 53% (*n*=78) of all facilities surveyed indicated that they distribute contraceptives. However, of the 83 non-Catholic facilities, 93% (*n*=77) do distribute contraceptives ([134] p 16).

In Timor Leste, clinics also lacked regular supplies of vaccines, which appeared to differentially impact on girls: girls 12–23 months were less likely to be fully immunised in comparison to boys in the same age range (23.4% compared to 29.8%). The gap increased in urban areas to 26% for girls and 40% for boys [73]. In rural areas, women needed to travel long distances to access reproductive technologies, and local midwives may not have received sufficient training to provide the range of modern methods of contraception [76].

Women’s caregiving role means that they were often responsible for taking children to clinics. Across the four contexts, shortages and stockouts were common [41, 89, 91, 138], and in Northern Uganda, researchers reported that these stockouts forced women to travel long distances to other public facilities or to sell assets to purchase medicine [138]. As most women across all four contexts are employed in the informal sector, their time off work directly translates into lost wages.

##### Medical products and technologies

We found little evidence of women’s differential vulnerability integrated into policies to ensure access to affordable medicines and technologies. One exception was global malaria protocols that prioritize the free provision of bednets to pregnant women, and prophylaxis treatment to reduce the impact of any potential malaria infection. Some research suggested that gender norms undermine the effectiveness of such progressive policies – with men taking bednets for their own use – while other studies suggested that higher risk behavior among men meant they are less likely to utilize bednets [139, 140]. The lack of clarity underscores the need for further study to examine the gendered dimensions of global policies that guide the distribution of medical products and technologies across different contexts.

To summarize, our review found insufficient research on gender equity within the ‘medical products and technology’ building block. The review did show that women suffered disproportionately from frequent shortages of medication and lack of medical equipment, due to the need for contraceptives, medications and equipment for antenatal and other reproductive health needs. Women also tended to experience the additional burden of trying to find medications for family members, and the associated time away from income-earning activities and household responsibilities. Yet engagement within this health system building block did not include an analysis of the differential vulnerabilities of women, nor address gender equity within its initiatives.

## Discussion: Key findings

### The challenge of health system engagement

All four contexts demonstrated the difficulties and challenges of rebuilding health systems in conflict affected and fragile contexts. International engagement in the health system struggled to achieve meaningful results and sustainably improve health outcomes for everyone - including men and women across age and other identity groups. For the purposes of our analysis, a key challenge of analyzing health systems is discerning the multiple factors that cause poor health outcomes. Are these health systems gender inequitable, or just weak? From a policy perspective, in a situation of constrained resources and with the focus on building national capacity, the answer to this question is important, and we have attempted to disentangle the differences between weak health systems and gender inequitable health systems within our analysis. Our review convincingly demonstrated that health system engagement is gender blind, with little effort to integrate gender analysis within health system programming, define gender equity within each health system component, or meaningfully and sustainably integrate gender into health system interventions.

### To what extent has post-conflict health system engagement met our benchmark of a gender equitable health system?

Table 4 synthesizes and presents our analysis against the key features of a gender equitable health system, identified in the methods section and taking forward Percival et al. 2014.

**Table 4:** Measuring Progress Against Gender Equitable Health System Attributes

Attributes of Gender Equitable Health System	Manifestation in Mozambique	Manifestation in Timor Leste	Manifestation in Sierra Leone	Manifestation in Northern Uganda
Provision: Health services addressing most urgent health care needs of men and women across life span in an appropriate manner.	While the delivery of health services has improved, adolescent girls and women lacked access to reproductive health care services. Concerns regarding respectful delivery.	Health service provision had improved dramatically, yet problematic health indicators for women and adolescent girls, particularly in rural areas.	Free health care initiative has prioritized care for women and children, although Ebola significantly weakened health system.	Health service delivery improved since war, yet significant shortfalls in service provision particularly for reproductive needs of women and girls. Concerns regarding respectful delivery.
Access: Ensure men and women across the life span are able to access and utilize services unimpeded by financial, social, geographic barriers.	Significant financial, geographic and cultural barriers existed for both men and women, while women faced the added burden of lack of autonomy over their health care decisions.	Barriers for health access existed, particularly geographic barriers.	Free health care initiative officially removed financial barriers, but research indicates that people still needed to pay out of pocket fees to secure care. Geographic barriers still exist as well as gender norms undermining women’s ability to make decisions.	Significant financial, geographic and cultural barriers to access, which were shaped by, gender norms.
Relevant, sex-disaggregated health information that informs policy.	Not consistently available.	Efforts to implement health information system with sex-disaggregated information underway.	Not consistently available, but planning is underway	Not consistently available.
Equitable opportunities for male and female health professionals working within the health system.	No strategy developed or implemented to promote gender equity in the human resources of the health system.	No strategy developed or implemented to promote gender equity in the human resources of the health system.	No strategy developed or implemented to promote gender equity in the human resources of the health system.	No strategy developed or implemented to promote gender equity in the human resources of the health system.
Equitable health outcomes among men and women and across age groups	HIV/AIDS rates 3–4 times higher among adolescent girls than boys; maternal health mortality among highest in the world.	Reproductive health outcomes for girls and women still problematic.	Double challenge from conflict and Ebola, health indicators significantly worsened, MMR particularly challenging.	Outcomes among women and adolescent girls remained problematic.

Following conflict, donor investments increased health care provision, although with sadly limited impacts on the health and well-being of women and adolescent girls. In all four contexts, access to services remains challenging for some groups, with geography and financial barriers, as well as gender norms and relationships within households, communities and the health sector limiting access, often in combination with each other. The regular use of sex-disaggregated health information to inform policy and practice is lacking. With respect to equal opportunities for female and male providers, none of the contexts had developed or implemented a strategy to promote female advancement in the human resources of the health system. This lack of gender equity impacted on health outcomes with continuingly problematic maternal and sexual and reproductive health outcome indicators for women and girls in all contexts.

As a result of our findings through this review of the literature, and our analysis of how health systems performed against the benchmarks of gender equity (outlined in Fig. 1), we suggest five key attributes of a ‘gender equitable health system’:

- Provides health care services that address the most urgent health care needs of men and women across the life span in an appropriate manner;
- Ensures men and women across the life span are able to access and utilize those services unimpeded by social, geographic and financial barriers;
- Produces relevant, sex disaggregated health information that informs policy;
- Provides equitable opportunities for male and female health professionals working within the health system; and
- Ensures equitable health outcomes among women and men, and across age groups.

As outlined in Table 4, our analysis shows that none of the four contexts of post-conflict health system engagement reflected these attributes of a gender equitable health system.

### Checking the gender box?

Our four contexts span both time and geography, with Mozambique’s war ending in 1992, while the violence in Northern Uganda ended in 2006. Over this time period, policy makers developed a greater awareness of the differential impact of conflict on women, girls, boys, and men, particularly the increased risk and incidence of sexual violence in conflict-affected states. This awareness, combined with the attention and resources devoted to the Millennium Development Goals (MDGs) and the SDGs meant that donors implemented a greater number of programs focused on sexual violence and maternal health in Timor Leste, Sierra Leone and Northern Uganda. However, as our analysis shows, there is little evidence that the increased resources resulted in sustained improvements in women’s health indicators – which underscores the extent that pernicious gender norms are replicated within the health system.

Instead of taking a comprehensive approach to women’s health, the increased resources tended to result in a narrow view of health and gender. The indicators related to women’s health, shaped by the MDGs, are antenatal care, attended deliveries, and maternal mortality. When donors and national stakeholders highlight their ‘gender-sensitive’ programs in post-conflict contexts, they immediately discuss efforts to stem maternal mortality or address sexual violence. With these programs, they have ‘checked’ the gender box. Yet our analysis shows that the significant donor assistance spent on maternal health, such as in Mozambique and Timor Leste, does not necessarily address the complex myriad of factors that contribute to poor maternal, sexual and reproductive health outcomes among women and girls. Analysis of how gender impacted on the performance of the health system and the differential health outcomes of men, women, boys and girls was not undertaken.

### What is the value-added of the framework of gender equitable health systems?

Despite the attention and resources of national governments, multilateral organizations and donors directed towards the health of women and girls, policymakers and researchers have not examined if and how the health system contributes to gender equity or inequity, or how gender inequity undermines the ability of health services to sustainably improve health outcomes. The focus of the global health community on ‘strengthening health systems’ has meant the dedication of more resources to them and efforts to improve their efficiency through reforms to financing methods, human resource allocation, and the organization of health services. More resources are necessary, but as our research demonstrates, money is insufficient to ensure sustainable and equitable health outcomes. Applying the benchmarks of gender equity to these four contexts demonstrates that pernicious gender norms within society are replicated within health systems, and that the failure to focus on gender equity in health system engagement undermines the effectiveness and efficiency of those systems. In the absence of efforts to improve gender equity within health systems, meaningful and sustained progress may remain stalled.

### Gender norms: Bricks and mortar

International health systems engagement is overwhelmingly focused on the ‘bricks’ of health systems – as is clear by WHO’s ‘building block’ nomenclature for the health system. And providing the ‘bricks’ is definitely critical for gender equitable health systems. Gender equitable health systems need skilled personnel, financing, and access to services, medicines and technologies - often referred to as the 'hardware' of health care.

But institutions such as health systems are embedded in and shaped by the software of health systems [10]: the interactions between people and systems, and within communities and families that are shaped by gender norms. While the concept of ‘software’ is useful, conceptually it refers to factors that ‘float’ around the building blocks. We instead utilize the term ‘mortar’ to indicate that these social norms hold the system together. Social and cultural beliefs about the role of men, women, girls and boys, how they should behave, how they should not behave, what life opportunities they should be afforded, and how they can and should be treated, form the mortar that cements these bricks together. In many lower income settings these social and cultural beliefs view women and girls as objects to dominate, limiting their life chances. The same beliefs shape the behaviour of men and boys — resulting in more violent, risk taking behavior. Such gender norms are harmful for everyone.

While providing the bricks of health systems is critical, for these systems to facilitate sustained improvement in the health outcomes of women and girls, international engagement should also pay attention to the mortar - gender norms. In all four contexts, the evidence suggests that sustained improvement in health outcomes will not occur without tackling gender norms that drive ill health. Yet health interventions do not undertake the gender analysis to understand these norms, and health systems place no value on ‘gender equity.’ In Mozambique, adolescent girls have an HIV prevalence rate 3–4 times that of their adolescent boy counterparts [141]. Among women of reproductive age in Mozambique, “there is a significant effect from the husband/partner’s health care decision making power on women’s intentions to use contraceptives, especially among rural women regardless of the number of living children” ([142] p. 7-8). Women report pressure to have children, and to maintain work responsibilities during pregnancy and even labour [106]. Evidence suggests only a third of pregnant women who face childbirth complications make the autonomous decision to see medical care – the rest wait for the views of their husband or his family [108]. Fifty-six percent of adolescent sexually active girls have unmet contraceptive needs [141]. Gender dynamics also impact on HIV positive women’s ability to decide when to have children, access health care services, or access treatment, including prevention of mother to child transmission (PMTCT) [143].

Sierra Leone and Northern Uganda mirror Mozambique in many ways. In all four contexts, women are often not treated with respect at health centres while cultural attitudes that surround sexuality, reproduction and child birth discourage health seeking behavior [41, 88]. The interaction between gender inequity and health outcomes is also clear in Timor Leste, where widespread acceptance of domestic violence coexists with high maternal mortality rates [40, 93, 124, 144].

As part of the efforts to improve health outcomes among girls and women, none of these four contexts included significant efforts to influence gender norms within health system interventions or to target men and address male behavior [142], although some studies highlighted the important of “engaging older male members such as community leaders and including discussion around gender norms and gender roles” [90]. Interventions that target and address gender norms have shown promise in decreasing rates of domestic violence [145], as well as improving anti-retroviral treatment among women [146–148], and accessing family planning [142]. Without changing these attitudes and behaviours, health interventions targeted towards men may make women more vulnerable to gender based violence [26, 149, 150].

### Health systems as promoting gender equity and equality? Who should take responsibility for engendering change in post-conflict contexts?

As noted above, the international community has a largely instrumental approach, with resources and reforms focused on the ‘bricks’ of health services. Donors and policy experts develop targets for key indicators – such as antenatal visits, attended births, and girls in school. This engagement is based on the belief that if those targets are met, the health system will be inherently gender equitable [151]. However, gender analysis has highlighted the importance of gender sensitive indicators to drive action and resist the ‘evaporation’ of a focus on gender to drive change [62]. Our review shows that we need action (and indicators) that go beyond the bricks of health systems to address the ‘mortar’: namely how gender roles and relations shape health care access, and impact on individuals’ experiences with the health care system (for example, do women experience respectful care and receive comprehensive access to reproductive health care).

This analysis raises difficult yet critical questions: to what extent is it the responsibility of health systems, as well as interventions in the health sector, to provide the mortar – to challenge subjects like gender and the cultural context of gender norms? And how can *international* engagement in these systems effectively tackle gender norms? The post-conflict period can create possibilities for change. Donor funds are plentiful, social norms are in flux, and there may be an appetite for political change [4]. The donor community on the whole is eager to fund projects to address specific issues – typically maternal and sexual and reproductive health – and as argued above, to check the gender box, but is largely reticent to tackle cultural and gender norms, and the gender inequities that drive certain health outcomes. Critiquing culture makes for difficult conversations with national stakeholders. Particularly given the time horizons of many donor programs, focusing on cultural change and the upstream social drivers of inequity may be perceived as risking the opportunity to generate less ambitious concrete outputs from projects and programs.

Throughout their life span, individuals interface with the health sector more than any other social institution. In addition, health care workers are respected members of their community who are accustomed to having difficult conversations about health determinants and outcomes [152]. If health care workers can tell men to stop smoking, they can tell them to respect the sexual and reproductive rights of girls and women. Risky behaviours can be confronted and society can cast a light on issues such as domestic and sexual violence, including the abuse of boys and men. There is little rationale for the health system not to broaden its scope to both reflect on its own role as a gendered institution/set of practices and engage with individuals and communities to discuss how gender norms impact on the health outcomes of both men and women. Gender transformative approaches would improve health systems and enable them to better respond to the needs of all members of the communities that they serve.

## Limitations

As outlined above, this is a new research area with limited available evidence and poor quality of data, making definitive conclusions challenging. More research into other case studies, particularly across diverse geographic areas, will be critical to further develop our findings regarding gender equity in health systems and how health systems can better address pernicious gender norms in society. Research into cases such as Rwanda and Liberia, where women are politically empowered, would enhance the evidence base on gender equity and health system reconstruction and reform. Recognizing these limitations, our systematic engagement with the available evidence only strengthened our conclusions regarding gender equity and health systems.

An important limitation to our research is the lack of available evidence on the differential burden of ill health faced by men. The majority of the evidence (including data) and analysis found through our review focuses on the relationship between women’s health and the health system. This poses important and challenging questions regarding gender and health system weakness, raised earlier: are health systems gender inequitable or are they simply weak? Do researchers focus on evidence related to women because of their reproductive role and the subsequent availability of health data on women, and thus conclude systems are gender inequitable when they are equally problematic for the health of men? Given the pervasive nature of gender inequities within most societies, and the evidence regarding the treatment of women within health systems presented through our analysis, we assume those gender inequities are reflected within the health system. But without data on men, this assumption is founded on evidence on women and girls, not on men and boys. There is a clear need to better identify how gender norms undermine male health outcomes and if and how health systems can better address this relationship. The experiences and health outcomes of people of other genders has also not been included; although these experiences are important, they are currently largely absent in policy documents, research and data sets.

The building block approach also poses limitations, as it focuses our interventions on specific elements of the health system, rather than the functioning of the system as a whole. It is a technocratic approach to health systems and does not include a sufficient focus on how health systems are embedded within the social context as well as the ‘software’ of health systems [10]. Moreover, it focuses our attention on the health system from the point of view of health workers and practitioners, rather than the point of view of patients. And as noted above, data sources in many of the contexts we examined were limited – relying on the Demographic Health Surveys and household surveys. In the absence of Central Vital Registration Systems, the validity and reliability of such data sources is uncertain.

## Conclusions

Our framework synthesis examined how gender considerations were integrated into development efforts to rebuild health systems within the post-conflict contexts of Mozambique, Sierra Leone, Timor Leste and Northern Uganda. Our review of the available evidence shows that health system engagement is gender blind, gender has not been sufficiently integrated into these interventions, and these health systems did not meet the benchmarks of gender equity within each building block or reflect the attributes of a gender equitable health system. Our review suggests that gender equitable health systems that address pernicious gender norms will support both stronger health systems and stronger societies. We look forward to further discussion and experience sharing on how the health sector can most meaningfully promote change within and beyond conflict-affected contexts.
